# Carbonic Anhydrase 2 and Na^+^/K^+^-ATPase Mediate Family-Dependent Nitrite Tolerance via Modulating Branchial Ion Transport and Acid–Base Balance in *Penaeus vannamei*

**DOI:** 10.3390/ani16111638

**Published:** 2026-05-27

**Authors:** Liping Zhou, Zhentao Ma, Xiuli Chen, Qingyun Liu, Yuliu Huang, Chunling Yang, Digang Zeng, Zhihong Zheng, Bin Zhang, Yueling Zhang, Yongzhen Zhao, Xianliang Zhao

**Affiliations:** 1 Guangdong Provincial Key Laboratory of Marine Biotechnology, Institute of Marine Sciences, Shantou University, Shantou 515063, China; 20lpzhou@stu.edu.cn (L.Z.); zhengzh@stu.edu.cn (Z.Z.); zhangyl@stu.edu.cn (Y.Z.); 2 Guangxi Laboratory on the Study of Coral Reefs in the South China Sea, School of Marine Sciences, Guangxi University, Nanning 530004, China; 15942391706@163.com; 3 Guangxi Key Laboratory of Aquatic Genetic Breeding and Healthy Aquaculture, Guangxi Academy of Fishery Sciences, Nanning 530021, China; chenxiuli2001@163.com (X.C.); liuqy198704@163.com (Q.L.); 15878757632@163.com (Y.H.); scsycl@163.com (C.Y.); zengdigang@126.com (D.Z.); zhangb41508@163.com (B.Z.)

**Keywords:** *Penaeus vannamei*, nitrite tolerance, ion transport, Na^+^-K^+^-ATPase, carbonic anhydrase 2

## Abstract

Nitrite is a key environmental challenge in intensive shrimp aquaculture, adversely affecting physiological regulation and survival. Although tolerant *Penaeus vannamei* families have been established by selective breeding, the basis of family-level variation in tolerance has yet to be clarified. In this study, nitrite-tolerant and nitrite-sensitive families were compared using survival analysis, transcriptomics, targeted qPCR validation, physiological assays, and RNA interference of representative transport-related genes. Under nitrite exposure, the tolerant family exhibited significantly higher survival and a distinct gill transcriptional response, characterized by stronger induction of acid–base and ion-transport genes, including carbonic anhydrase 2 (*CA2*), the Na^+^/K^+^-ATPase subunits *ATP1A* and *ATP1B*, as well as several V-type H^+^-ATPase-related genes. These transcriptional changes were accompanied by elevated ATP content and Na^+^/K^+^-ATPase activity, improved hemolymph pH stability, and reduced nitrite accumulation in both gill and hemolymph. RNAi-mediated knockdown of *CA2* or *ATP1B* attenuated the nitrite-induced transport response, decreased ATP content and NKA activity, exacerbated hemolymph acidification, promoted internal nitrite accumulation, and ultimately reduced shrimp survival under nitrite stress. Family-based validation further showed that the tolerant family displayed higher survival than the sensitive family in the dsEGFP group, whereas this advantage was markedly reduced after *CA2* or *ATP1B* knockdown under nitrite stress. These findings highlight that strengthened branchial ion transport and acid–base regulation represent key physiological mechanisms underlying nitrite tolerance in resistant shrimp families.

## Simple Summary

Nitrite toxicity is a major threat to shrimp health and aquaculture production. In this study, we combined transcriptomic, physiological, and RNA interference analyses to characterize the basis of nitrite tolerance in *Penaeus vannamei*. We found that nitrite-tolerant shrimp maintain stronger gill ion transport and acid–base balance under nitrite stress. Key genes, including carbonic anhydrase 2 (*CA2*) and Na^+^/K^+^-ATPase subunits (*ATP1A*, *ATP1B*), were more highly activated in tolerant shrimp, accompanied by higher ATP levels, Na^+^/K^+^-ATPase activity, and stable hemolymph pH. Knockdown of *CA2* or *ATP1B* impaired ion transport, aggravated acidification, and reduced survival. Our results reveal that enhanced gill homeostasis via CA2-mediated ion regulation is a key mechanism of nitrite tolerance. These findings provide new targets for breeding nitrite-resistant shrimp and improving disease control strategies in aquaculture.

## 1. Introduction

For decades, nitrite accumulation has been recognized as a recurrent constraint in intensive shrimp aquaculture, especially when nitrification becomes unstable under high feeding intensity and biomass loading [1,2,3,4]. In intensive aquaculture and recirculating systems, nitrite concentrations may rise well above safe levels, and *P. vannamei* is considered especially sensitive to nitrite exposure [5,6,7,8]. Nitrite is taken up across epithelial surfaces, especially the gill, and can severely impair respiratory function and physiological homeostasis in aquatic animals [9,10,11,12]. This toxicological framework has been well established in teleosts, in which gill nitrite uptake compromises blood oxygen transport and induces a hypoxia-like internal state [13,14,15,16,17]. A comparable physiological consequence is thought to occur in decapod crustaceans, although the oxygen carrier differs. Whereas fish develop methemoglobinemia through hemoglobin oxidation, penaeid shrimp rely on hemocyanin, whose oxidation by nitrite reduces oxygen-binding capacity and contributes to tissue hypoxia [18,19,20]. Collectively, these observations indicate that nitrite exposure ultimately converges on functional hypoxia and systemic homeostatic disturbance, despite differences in the immediate biochemical targets among aquatic taxa [13,21,22,23].

Nitrite toxicity is now understood as a context-dependent physiological challenge in aquatic animals. Salinity and chloride availability can markedly influence nitrite uptake and toxicity, consistent with competition at chloride-linked epithelial transport pathways and altered electrochemical conditions [24,25]. Evidence from decapod crustaceans further indicates that external chloride is closely associated with mortality, ion regulation, and acid–base balance during nitrite exposure, highlighting that nitrite entry and homeostatic regulation are mechanistically coupled rather than independent processes [26,27,28]. In crustaceans, the gill is a key tissue for nitrite processing and serves as the principal epithelial organ for ionic and acid–base regulation [29,30,31]. Because nitrite uptake is closely linked to epithelial anion exchange and systemic acid–base status, variation in gill transport and buffering capacity may critically influence internal nitrite burden and tolerance phenotype [32]. This view is further supported by recent evidence in *P. vannamei* showing that branchial anion exchanger 2 and Na^+^/K^+^/2Cl^−^ Cotransporter (NKCC) 1 contribute to nitrite handling, thereby linking epithelial transport activity to internal nitrite accumulation during nitrite exposure [33]. Meanwhile, enhanced gill ion-transport capacity, carbonic anhydrase-associated buffering, and energy-supported homeostatic regulation have been increasingly associated with improved stress tolerance in shrimp and other crustaceans [34,35]. Accordingly, understanding how gill transport and buffering systems respond to nitrite stress is central to resolving the physiological basis of tolerance.

Family-based designs provide a powerful framework for dissecting the mechanistic basis of nitrite tolerance while generating knowledge of direct relevance to selective breeding. Recent family-based breeding efforts have further shown that nitrite tolerance in shrimp is a heritable trait and that nitrite-tolerant families with stable phenotypic divergence can be obtained through selective breeding, providing an important foundation for the mechanistic dissection of tolerance formation [36]. Family-based designs provide a powerful framework for dissecting the mechanistic basis of nitrite tolerance while generating knowledge of direct relevance to selective breeding. In *P. vannamei*, integrated physiological, transcriptomic, and metabolomic analyses have revealed that nitrite tolerance is associated with coordinated reprogramming of energy metabolism, oxidative stress responses, and homeostatic regulation [37,38]. Gill transcriptome comparison between the two families further revealed marked divergence in molecular responses to nitrite exposure. Consistent with this observation, our previous study demonstrated that nitrite tolerance is associated with differential regulation of autophagy and apoptosis [23]. Together with the genetic evidence that nitrite tolerance is linked to identifiable quantitative trait loci [36], these studies support the existence of family-dependent tolerance divergence, but they do not explain whether this divergence is functionally linked to differences in gill ion transport and acid–base buffering capacity [23,36]. In particular, it remains unclear whether tolerant families can better restrict internal nitrite accumulation by maintaining stronger gill homeostatic regulation during nitrite exposure. It also remains unresolved which core transport-related genes directly contribute to this homeostatic advantage and ultimately determine tolerance phenotype.

In the present study, two *Penaeus vannamei* families with contrasting nitrite tolerance were examined through survival analysis, gill transcriptomics, targeted qPCR validation, physiological assays, and RNA interference of representative transport-related genes. We hypothesized that the tolerant family would possess a stronger branchial capacity to maintain ion transport and acid–base homeostasis under acute nitrite stress, thereby limiting internal nitrite accumulation and improving survival. To test this hypothesis, we compared survival and gill transcriptional responses between the two families during nitrite exposure, focusing on transport- and acid–base-related pathways, and further assessed the functional roles of *CA2* and *ATP1B*. The tolerant family exhibited stronger gill transport and buffering responses, lower internal nitrite accumulation, and higher survival, whereas disruption of either factor impaired physiological homeostasis and reduced tolerance. Together, these findings indicate that carbonic anhydrase-associated branchial ion transport and acid–base regulation are central to family-dependent nitrite tolerance in *Penaeus vannamei* and provide mechanistic support for selective breeding of nitrite-tolerant shrimp lines.

## 2. Materials and Methods

### 2.1. Ethics Statement

All shrimp breeding, handling, and sampling procedures were approved by the Animal Care and Ethics Committee of Guangxi Academy of Fishery Sciences (2025-GAFS-09) and complied with relevant ethical guidelines.

### 2.2. Animals and Treatment

Pacific white shrimp were obtained from the family-based breeding population maintained at the Guangxi Academy of Fishery Sciences Shrimp Breeding Center. Based on multi-generational breeding records, one nitrite-tolerant family and one nitrite-sensitive family were selected. Prior to the trial, shrimp with similar body weight (5.0–8.0 g) were acclimated for 3 days in 400-L aerated tanks (Dongguan Rongcheng Plastic Products Co., Ltd., Dongguan, China). Water quality was maintained at 25‰ salinity, pH 7.9 ± 0.1, 27 ± 0.5 °C, and dissolved oxygen at 5.5–6.5 mg/L. Throughout the experiment, shrimp were fed twice daily at 09:00 and 20:00 with a commercial pelleted diet. The feed was supplied by Fujian Tianma Technology Group Co., Ltd. (Fuzhou, China) and contained 38% crude protein, 4% crude lipid, 8% crude fiber, 16% ash, and 11% moisture. Uneaten feed and feces were removed regularly to maintain stable rearing conditions.

### 2.3. Nitrite Exposure and Experimental Design

A preliminary acute nitrite exposure assay was first performed to estimate the median lethal concentration (LC_50_) of the nitrite-sensitive and nitrite-tolerant families at different time points. Based on the LC_50_ results, the nitrite challenge condition used in the subsequent experiment was established by adding sodium nitrite (NaNO_2_) to the culture water to a final concentration of 600 mg/L. To keep the exposure level stable during the experiment, the rearing water was replaced every 24 h, followed by re-addition of NaNO_2_ to restore the desired level. Shrimp from the control group were maintained under the same rearing conditions but without nitrite supplementation.

Shrimp were assigned to four groups: sensitive control (SC), tolerant control (TC), sensitive under nitrite stress (SN), and tolerant under nitrite stress (TN). Each group was conducted in three independent tanks, with 40 shrimp per tank. Survival was recorded at 0, 24, 48, 72, and 96 h after exposure. Individuals that remained immobile and unresponsive to gentle stimulation were recorded as dead. Survival curves were analyzed using the Kaplan–Meier method, and group differences were tested with the log-rank (Mantel–Cox) test.

### 2.4. Sampling and Tissue Processing

Following 72 h of nitrite challenge, gill samples were collected for transcriptome sequencing and qPCR verification, while hemolymph was simultaneously harvested for pH determination and nitrite concentration analysis. Gill tissues were dissected on ice, gently washed with sterile PBS buffer (140 mM NaCl, 2 mM NaH_2_PO_4_, 10 mM Na_2_HPO_4_, pH 7.4), immediately frozen in liquid nitrogen, and kept at −80 °C until analysis.

Hemolymph was immediately withdrawn from the abdominal sinus using a sterile syringe containing anticoagulant buffer composed of 26 mmol/L sodium citrate, 30 mmol/L citric acid, 100 mmol/L glucose, and 140 mmol/L NaCl.

### 2.5. Hemolymph pH and Nitrite Accumulation

Hemolymph pH was measured immediately after collection at the experimental temperature using a micro pH electrode calibrated with standard buffer solutions. For hemolymph pH, hemolymph nitrite, and gill nitrite measurements, three biological replicates were analyzed for each group at each time point, with each replicate consisting of pooled samples from three shrimp. Nitrite concentrations in hemolymph and gill homogenates were quantified with a nitrite assay kit from Nanjing Jiancheng Bioengineering Institute (Nanjing, China) in accordance with the supplier’s instructions. For gill analysis, tissues were homogenized in ice-cold PBS and centrifuged at 12,000× *g* for 10 min at 4 °C, after which the supernatant was collected for nitrite quantification.

### 2.6. Histological Analysis

Gill tissues were collected and fixed in 4% paraformaldehyde for 24 h, followed by dehydration in a graded ethanol series comprising 80% for 45 min, 90% for 45 min, 95% for 30 min, and 100% ethanol for 30 min. The dehydrated tissues were cleared with xylene, embedded in paraffin, sectioned at 5 μm, and stained with hematoxylin and eosin. Sections were examined under a bright-field microscope (ECLIPSE 90i, Nikon, Tokyo, Japan). Gill histopathological alterations were semi-quantitatively scored based on filament deformation, epithelial vacuolation, and hemolymph sinus congestion. Each parameter was graded on a 0–3 scale, where 0 = no obvious lesion, 1 = mild, 2 = moderate, and 3 = severe.

### 2.7. Na^+^/K^+^-ATPase (NKA) Activity and ATP Content Assays

Gill supernatants prepared according to the procedure described in Section 2.5 were used for the determination of enzymatic activity and ATP content. Three biological replicates were analyzed for each group at each time point, with each replicate consisting of pooled samples from three shrimp. Protein concentration was determined with a BCA Protein Assay Kit (GenStar, Beijing, China). Na^+^/K^+^-ATPase activity was analyzed using a kit from Nanjing Jiancheng Bioengineering Institute (Nanjing, China), and ATP levels were determined with an ATP Detection Kit (Beyotime Biotechnology, Shanghai, China) according to the manufacturers’ instructions. The measured NKA activity and ATP content were normalized to total protein concentration.

### 2.8. RNA Extraction and qPCR Validation

Total RNA was extracted from gill tissue using TRNzol Universal (TIANGEN, Beijing, China). RNA yield and quality were assessed with a NanoDrop 2000 spectrophotometer (Thermo Fisher Scientific, Waltham, MA, USA) based on the A260/280 and A260/230 ratios, and RNA integrity was verified by agarose gel electrophoresis. First-strand cDNA was generated using the PrimeScript™ RT Reagent Kit (Perfect Real Time) (Takara, Beijing, China). Quantitative real-time PCR was carried out with SYBR Green Master Mix (GenStar, Beijing, China) on a QuantStudio 3 system (Applied Biosystems, Foster City, CA, USA). Primer sequences are provided in Table A1, and transcript levels were calculated by the 2^−ΔΔCt^ method with *β-actin* (GenBank accession: LC382464.1) as the reference gene [39]. Each assay included technical triplicates and at least three independent biological replicates.

### 2.9. Transcriptome Sequencing and RNA-Seq Analysis

Gill tissues from the SC, TC, SN, and TN groups were subjected to RNA-seq analysis. Libraries were constructed with the TruSeq™ RNA Sample Prep Kit (Illumina, San Diego, CA, USA) and sequenced in paired-end mode on the Illumina NovaSeq 6000 by Majorbio Bio-pharm Technology Co., Ltd. (Shanghai, China). Briefly, poly(A)+ RNA was isolated from total RNA with oligo(dT) magnetic beads, followed by cDNA synthesis using random primers to obtain double-stranded cDNA [40,41]. After end repair, adapter ligation, and PCR amplification, qualified libraries were sequenced.

Raw reads were processed with Trimmomatic (v0.36) for quality filtering, and the clean reads were aligned to the *P. vannamei* reference genome (ASM378908v1; NCBI) with HISAT2 (v2.2.1) under default parameters [42,43]. Aligned reads were assembled with Cufflinks (v2.2.1), and the resulting transcripts or unigenes were annotated against the Pfam, NR, NT, COG, GO, and KEGG databases. Transcript abundance was normalized as transcripts per million (TPM) [44]. Differentially expressed genes (DEGs) were identified with DESeq2 (v1.24.0), and significance was adjusted by the Benjamini–Hochberg false discovery rate procedure. Genes meeting the thresholds of adjusted *p* < 0.05 and |fold change| > 2.0 were defined as DEGs. KEGG pathway enrichment of DEGs was performed with clusterProfiler (v4.0) [45]. In addition, principal component analysis (PCA) was conducted to evaluate global sample separation and the reproducibility among biological replicates, whereas Venn analysis was used to identify common and comparison-specific DEGs in the SN vs. SC and TN vs. TC groups. Representative DEGs were subsequently selected for qPCR validation, and the corresponding primer sequences are listed in Table A1.

### 2.10. dsRNA Synthesis and RNA Interference

Double-stranded RNA (dsRNA) targeting *CA2* and *ATP1B* (designated as dsCA2 and dsATP1B, respectively) was in vitro synthesized with the HiScribe T7 Rapid High-Yield RNA Synthesis Kit (New England Biolabs, Ipswich, MA, USA, Cat. No. E2050S), and dsEGFP was used as the negative control. Shrimp were randomly assigned to three groups and intramuscularly injected with 100 μL of dsRNA solution containing dsEGFP, dsCA2, or dsATP1B (100 ng/μL; 10 μg per shrimp). dsEGFP served as the negative control. At 24 h after injection, all shrimp were subjected to 600 mg/L NaNO_2_ exposure. Gill tissues and hemolymph were sampled at 24, 48, and 72 h post-nitrite exposure to determine NKA activity, hemolymph pH, and nitrite accumulation, while the knockdown efficiency of *CA2* and *ATP1B* genes was verified by qPCR. Primer sequences used in this assay are provided in Table A1.

### 2.11. Statistical Analysis

Results are shown as mean ± SD based on three independent biological replicates. Prior to parametric analysis, normality and homogeneity of variance were evaluated using the Shapiro–Wilk test and Levene’s test, respectively. Survival differences were assessed using the log-rank (Mantel–Cox) test. Comparisons between two groups were performed using a two-tailed Student’s *t*-test, whereas datasets involving two factors were analyzed using ordinary two-way ANOVA followed by Tukey’s multiple comparisons test. Pearson correlation analysis was used to assess the consistency between RNA-seq and RT-qPCR data. All statistical analyses were conducted in SPSS 25.0, and figures were prepared in GraphPad Prism v8.0.2. Statistical significance was set at *p* < 0.05.

## 3. Results

### 3.1. Family-Dependent Differences in Survival, Nitrite Accumulation, and Gill Histology Under Nitrite Stress

**Figure 1:**
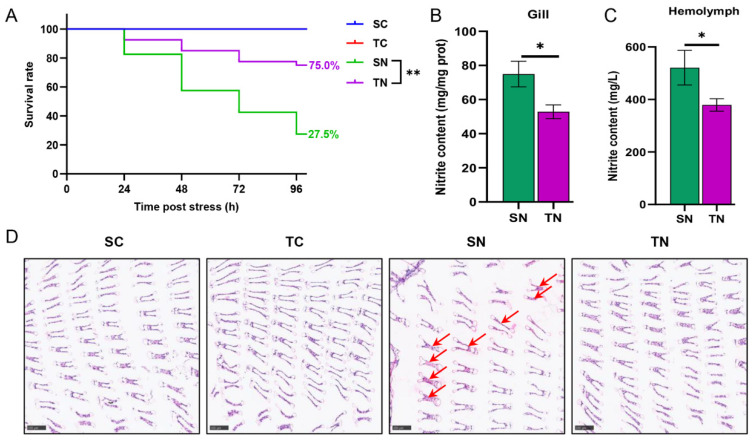
Comparative survival, nitrite accumulation, and gill histology between nitrite-sensitive and -tolerant shrimp families. (**A**) Survival curves of nitrite-sensitive and nitrite-tolerant *P. vannamei* families under control and nitrite stress conditions for 96 h. Survival differences were assessed using the log-rank (Mantel–Cox) test. (**B**,**C**) Nitrite content in the gill and hemolymph of the two families at 72 h after nitrite exposure. Data are presented as mean ± SD. Statistical difference was evaluated using Student’s *t*-test. Asterisks indicate significant differences between families. *p* < 0.05 (*), and *p* < 0.01 (**). (**D**) Representative H&E-stained gill sections of SC, TC, SN, and TN groups. Red arrows indicate gill lesions in the sensitive family under nitrite stress. Scale bars = 100 μm. SC, sensitive family control; SN, sensitive family under nitrite stress; TC, tolerant family control; TN, tolerant family under nitrite stress.

As an initial assessment of family-dependent nitrite tolerance, LC_50_ values were estimated at different time points after nitrite exposure. The nitrite-tolerant family consistently showed higher LC_50_ values than the nitrite-sensitive family, reaching 1119.57 vs. 1057.93 mg/L at 24 h, 990.80 vs. 729.46 mg/L at 48 h, 788.45 vs. 529.67 mg/L at 72 h, and 689.84 vs. 349.81 mg/L at 96 h (Figure S1). Consistently, during the 96 h nitrite challenge, both families maintained 100% survival under control conditions, whereas survival under nitrite stress declined to 27.5% in the sensitive family and 75.0% in the tolerant family at 96 h (Figure 1A). At 72 h after nitrite exposure, nitrite concentrations in both the gill and hemolymph were significantly higher in the sensitive family than in the tolerant family, reaching 74.9 vs. 52.8 mg/L in the gill and 521.2 vs. 379.1 mg/L in the hemolymph, respectively (Figure 1B,C). This pattern indicates that the tolerant family was more effective in limiting internal nitrite accumulation during nitrite stress. Representative histological observations suggested that nitrite exposure caused more evident gill lesions in the sensitive family, including filament deformation, epithelial vacuolation, and hemolymph sinus congestion, whereas the tolerant family retained a comparatively more intact gill structure (Figure 1D). Together, these results indicate that the tolerant family displayed enhanced nitrite tolerance to nitrite stress, possibly owing to its reduced nitrite accumulation in vivo.

### 3.2. Transcriptomic Profiling of Nitrite-Sensitive and Nitrite-Tolerant Families Under Nitrite Exposure

**Figure 2:**
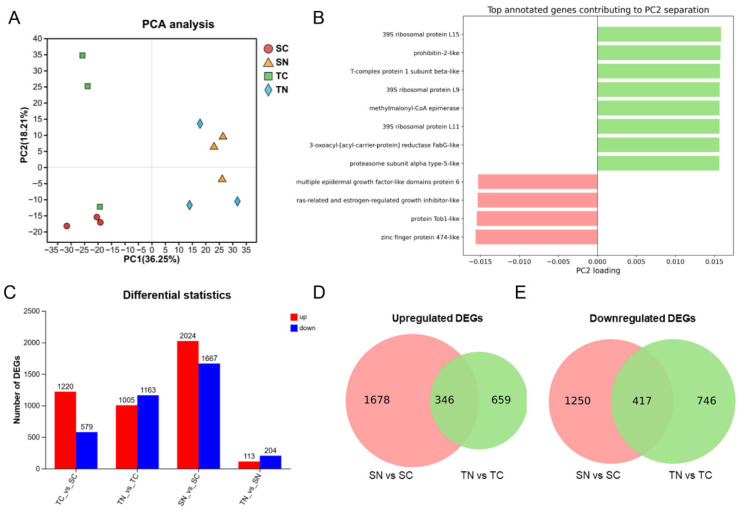
Transcriptomic responses of nitrite-sensitive and nitrite-tolerant families to nitrite stress. (**A**) PCA of gill transcriptomes from nitrite-sensitive and nitrite-tolerant *P. vannamei* families under control and nitrite stress conditions. SC, sensitive family control; SN, sensitive family under nitrite stress; TC, tolerant family control; TN, tolerant family under nitrite stress. (**B**) Top annotated genes contributing to PC2 separation. (**C**) Numbers of upregulated and downregulated DEGs in the indicated comparisons. (**D**) Venn diagram showing shared and family-specific upregulated DEGs in response to nitrite stress in the sensitive and tolerant families. (**E**) Venn diagram showing shared and family-specific downregulated DEGs in response to nitrite stress in the sensitive and tolerant families.

To characterize the transcriptional features associated with family-specific variation in nitrite tolerance, gill transcriptomes were compared across sensitive and tolerant families under basal and nitrite-challenged conditions. PCA showed clear separation among the four groups, with PC1 mainly distinguishing samples before and after nitrite stress and PC2 separating the SC and TC groups, indicating significant differences in transcriptomic profiles among groups (Figure 2A). Consistent with this pattern, genes contributing strongly to PC2 separation included several annotated transcripts related to ribosomal function, protein folding, mitochondrial regulation, and growth-associated signaling, further supporting the existence of pre-existing family-specific differences under basal conditions (Figure 2B). Among these contributors, prohibitin-2 and 39S ribosomal proteins L15 and L9 were notable. Prohibitin-2 is associated with mitochondrial maintenance, whereas 39S ribosomal proteins are involved in mitochondrial translation. Because mitochondrial activity and protein synthesis are closely linked to ATP production and protein renewal, these PC2-associated genes suggested basal differences in mitochondrial and translational status between the two families. Differential expression analysis showed that the sensitive family exhibited a substantially larger DEG set than the tolerant family under nitrite stress, indicating a stronger overall transcriptional perturbation (Figure 2C; Tables S1–S4). For downregulated genes, 417 were shared, while 1250 and 746 were specific to the sensitive and tolerant families, respectively (Figure 2D).

**Figure 3:**
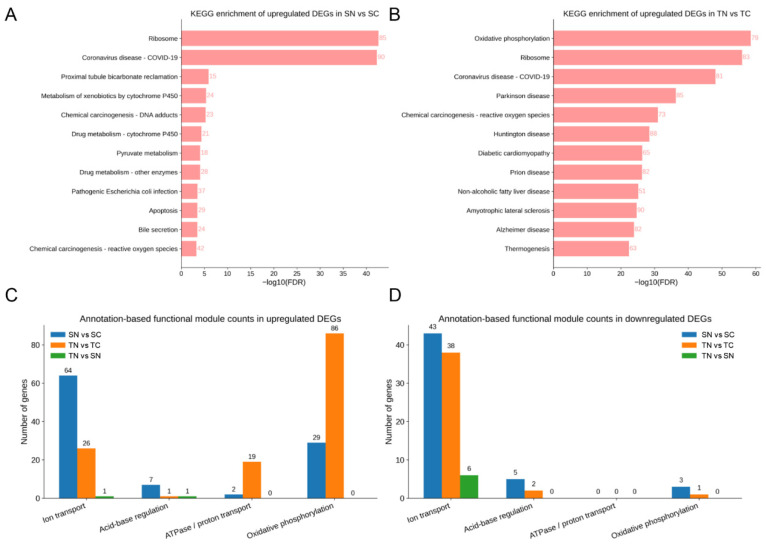
KEGG enrichment and functional module classification of DEGs associated with nitrite stress and family-dependent tolerance in *P. vannamei*. (**A**) Upregulated DEGs in SN vs. SC. (**B**) Upregulated DEGs in TN vs. TC. (**C**) Functional module counts in upregulated DEGs. (**D**) Functional module counts in downregulated DEGs. SC, sensitive family control; SN, sensitive family under nitrite stress; TC, tolerant family control; TN, tolerant family under nitrite stress.

To further characterize the biological pathways underlying the distinct transcriptional responses of the two families to nitrite stress, KEGG enrichment was conducted for DEG sets derived from both within-family and cross-family comparisons. In the sensitive family, upregulated DEGs were mainly enriched in ribosome, proximal tubule bicarbonate reclamation, pyruvate metabolism, metabolism of xenobiotics by cytochrome P450, drug metabolism, apoptosis, and reactive oxygen species-related pathways (Figure 3A), indicating a broad stress-responsive adjustment involving metabolic and acid–base-associated processes. Among the downregulated DEGs in the same comparison, the enriched pathways were mainly related to transporter function, glycan biosynthesis, nitrogen metabolism, lipid metabolism, and amino acid/tRNA-associated processes, together with several annotated categories linked to DNA damage and structural stress responses (Figure S2A), suggesting suppression of transport- and metabolism-related processes in the sensitive family under nitrite stress. By contrast, upregulated DEGs in the tolerant family were mainly enriched in oxidative phosphorylation, ribosome, thermogenesis, and other mitochondria- and energy metabolism-related pathways, with several annotated disease-related KEGG categories largely converging on mitochondrial respiratory chain, oxidative stress, and energy production modules (Figure 3B). This enrichment pattern was consistent with the PC2-associated basal differences described above. In particular, oxidative phosphorylation enrichment was in line with the involvement of prohibitin-2-like, whereas ribosome-related enrichment was consistent with the contribution of 39S ribosomal proteins L15 and L9. Together, these results suggest that the tolerant family preferentially maintained mitochondrial energy metabolism and protein-synthesis-related processes during nitrite exposure, which may support ion transport and acid–base homeostasis under stress. The downregulated DEGs in the tolerant family were mainly enriched in glycosaminoglycan biosynthesis, cell adhesion molecules, phospholipase D signaling, Ras signaling, focal adhesion, glycerolipid metabolism, and glycerophospholipid metabolism, together with other signaling- and structure-related categories (Figure S2B), indicating a distinct transcriptional adjustment pattern from that observed in the sensitive family. Further comparison between the two stressed families showed that genes expressed at higher levels in the tolerant family were enriched in pancreatic secretion, gastric acid secretion, parathyroid hormone synthesis, secretion and action, phosphatidylinositol signaling, arginine and proline metabolism, vascular smooth muscle contraction, and chemokine signaling pathways (Figure S2C), supporting differences in secretion-related regulation, signaling, and metabolic adaptation between the two families. Conversely, genes expressed at lower levels in the tolerant family than in the sensitive family under nitrite stress were enriched mainly in immune- and inflammation-related pathways, apoptosis-related pathways, purine metabolism, steroid hormone biosynthesis, and several pathogen-response annotated categories (Figure S2D). Annotation-based functional module analysis further supported these pathway-level differences, showing that nitrite-responsive genes in the sensitive family were more frequently assigned to ion transport and acid–base regulation, whereas those in the tolerant family were more strongly represented in ATPase/proton transport and oxidative phosphorylation modules (Figure 3C). Similar transport- and metabolism-related trends were also reflected in the downregulated modules (Figure 3D). Together, these results indicate that the family-dependent transcriptional divergence under nitrite stress was mainly centered on ion transport, acid–base regulation, ATPase/proton transport, and oxidative phosphorylation.

### 3.3. Comparative Transcriptomic Analysis Reveals Transport- and Acid–Base-Related Mechanisms Linked to Nitrite Tolerance

**Figure 4:**
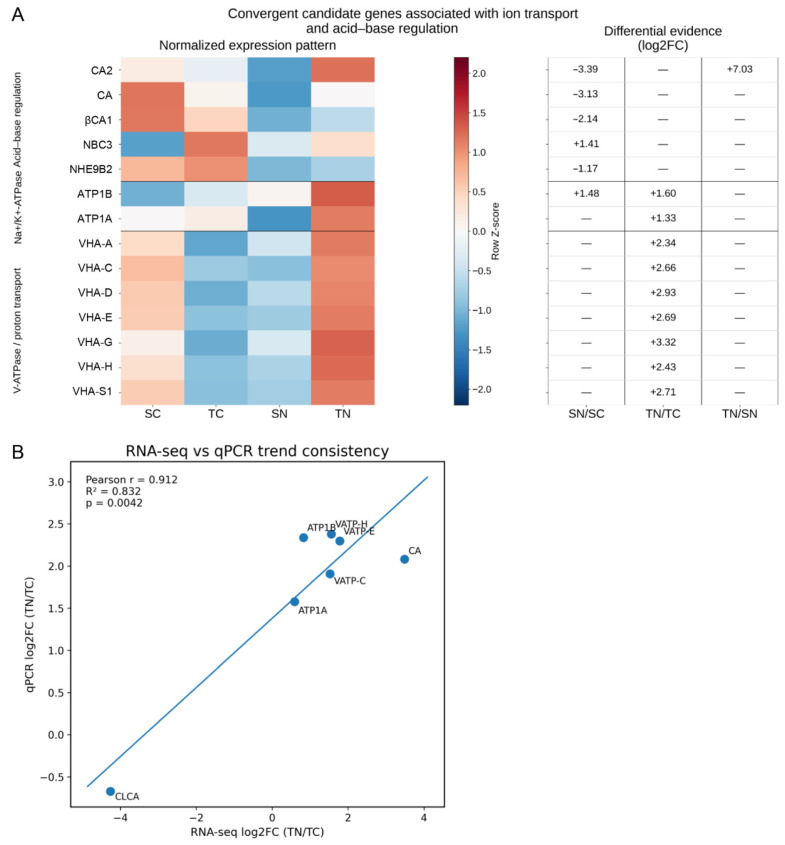
Candidate genes associated with ion transport and acid–base regulation and their validation by RT-qPCR. (**A**) Heatmap showing the normalized expression patterns of convergent candidate genes related to acid–base regulation, Na^+^/K^+^-ATPase, and V-ATPase/proton transport in SC, TC, SN, and TN groups, together with their differential expression evidence in the indicated comparisons. (**B**) Correlation analysis between RNA-seq and RT-qPCR results for selected candidate genes in the TN/TC comparison. Pearson correlation analysis showed a significant positive correlation between the two methods (r = 0.912, R^2^ = 0.832, *p* = 0.0042). SC, sensitive family control; SN, sensitive family under nitrite stress; TC, tolerant family control; TN, tolerant family under nitrite stress.

Among the DEGs enriched in the four functionally relevant pathways identified above, several candidates related to branchial ion transport and acid–base homeostasis were highlighted across the four groups, including *CA2*, *ATP1A*, *ATP1B*, and multiple V-ATPase subunits involved in proton transport (Figure 4A). Several carbonic anhydrase-related genes displayed family-dependent expression patterns, with *CA* and *βCA1* being downregulated in the sensitive family after nitrite exposure, whereas *CA2* remained more highly expressed in the tolerant family under stress. In contrast, *ATP1A*, *ATP1B*, and multiple V-ATPase subunits were coordinately induced in the tolerant family, indicating a stronger ATP-dependent transport and proton-regulatory response. By comparison, the sensitive family showed weaker activation of these transport-related genes. qPCR validation of seven representative genes further confirmed the RNA-seq trends, showing a strong positive correlation between the two datasets (Figure 4B). Together, these findings identify genes related to *CA2*, Na^+^/K^+^-ATPase, and V-ATPase as key candidates associated with family-dependent nitrite tolerance.

### 3.4. Expression and Physiological Changes in CA2 and Na^+^/K^+^-ATPase Genes Under Nitrite Stress

**Figure 5:**
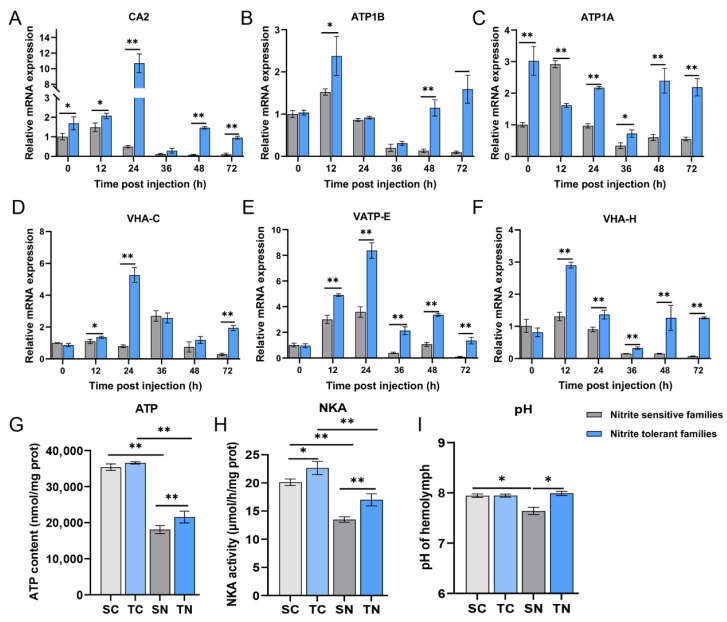
Relative mRNA expression of transport-related genes and physiological differences between nitrite-sensitive and -tolerant families under nitrite stress. (**A**–**F**) Relative mRNA expression of *CA2* (**A**), *ATP1B* (**B**), *ATP1A* (**C**), *VHA*-C (**D**), *VHA-E* (**E**), and *VHA-H* (**F**) in the gills of the nitrite-sensitive and nitrite-tolerant families at different time points after nitrite exposure. (**G**–**I**) ATP content (**G**), Na^+^/K^+^-ATPase activity (**H**), and hemolymph pH (**I**) of the two families at 72 h after nitrite stress. Gray bars indicate the nitrite-sensitive family, and blue bars indicate the nitrite-tolerant family. SC, sensitive family control; SN, sensitive family under nitrite stress; TC, tolerant family control; TN, tolerant family under nitrite stress. Data are presented as mean ± SD (*n* = 3). Statistical differences were analyzed using ordinary two-way ANOVA followed by Tukey’s multiple comparisons test. Asterisks indicate significant differences between the indicated groups. * *p* < 0.05, ** *p* < 0.01.

To further examine family-dependent differences in the dynamic expression of representative ion transport- and acid–base-related genes under nitrite stress, their temporal patterns in the gill were analyzed. Overall, the two families exhibited distinct transcriptional dynamics during nitrite exposure, with the tolerant family generally showing stronger or more sustained induction of several key genes. *CA2* showed a rapid response in the tolerant family and peaked at 24 h with a 10.7-fold increase (*p* < 0.01), whereas its expression in the sensitive family remained low (≤1.5-fold) and gradually declined thereafter (Figure 5A). *ATP1B* displayed a delayed induction pattern. In the sensitive family, its expression increased to 1.5-fold at 12 h and then declined progressively to 0.1-fold by 72 h, whereas in the tolerant family it peaked at 2.4-fold at 12 h, decreased at 24–36 h, and then increased again to 1.2-fold at 48 h and 1.6-fold at 72 h (*p* < 0.01) (Figure 5B). *ATP1A* also exhibited clear family-dependent dynamics. Its expression was transiently higher in the sensitive family at 12 h (2.9-fold vs. 1.6-fold) but remained significantly higher in the tolerant family at 24, 48, and 72 h, reaching 2.2-, 2.4-, and 2.2-fold, respectively (*p* < 0.01) (Figure 5C). Similarly, several genes involved in acid–base regulation, including V-type H^+^-transporting ATPase subunit C (*VHA-C*), subunit E (*VHA-E*), and subunit H (*VHA-H*), were more strongly induced in the tolerant family, particularly at 12–24 h after nitrite exposure, with *VHA-E* reaching 4.9-fold at 12 h (*p* < 0.01) (Figure 5D–F). Consistent with these transcriptional patterns, at 72 h after nitrite stress, the tolerant family maintained significantly higher ATP content (21,557.0 vs. 18,089.9 nmol/mg protein, *p* < 0.01), NKA activity (17.1 vs. 13.5 μmol/h/mg protein, *p* < 0.01), and hemolymph pH (8.0 vs. 7.6, *p* < 0.05) than the sensitive family (Figure 5G–I), indicating better maintenance of transport-related physiological homeostasis under nitrite stress. The coordinated upregulation of *CA2* and Na^+^/K^+^-ATPase genes, together with improved ATP supply, elevated NKA activity, and stabilized hemolymph pH, collectively enhances gill physiological homeostasis and reduces nitrite accumulation in vivo. These findings highlight that strengthened branchial ion transport and acid–base regulation represent key physiological mechanisms underlying nitrite tolerance in resistant shrimp families.

### 3.5. CA2-Mediated Ion Transport and Acid–Base Regulation Under Nitrite Stress

**Figure 6:**
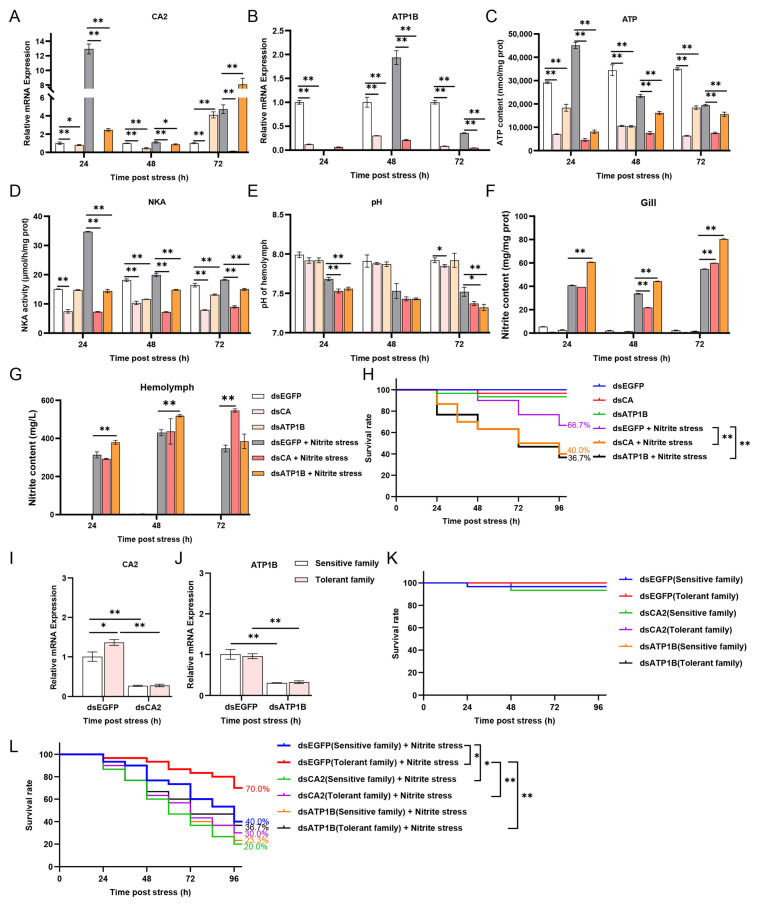
Functional validation of CA2 and ATP1B in nitrite tolerance of *P. vannamei*. (**A**,**B**) Relative mRNA expression of *CA2* and *ATP1B* after dsRNA injection. (**C**–**E**) ATP content, NKA activity, and hemolymph pH after gene silencing with or without nitrite stress. (**F**,**G**) Nitrite content in the gill and hemolymph under different treatments. (**H**) Survival curves of shrimp after *CA2* or *ATP1B* knockdown during a 96 h nitrite challenge. dsEGFP, dsRNA control; dsCA2, *CA2* knockdown; dsATP1B, *ATP1B* knockdown. (**I**,**J**) Knockdown efficiency of *CA2* and *ATP1B* in the sensitive and tolerant families, respectively. (**K**) Survival curves of the sensitive and tolerant families after *CA2* or *ATP1B* knockdown under control conditions. (**L**) Survival curves of the sensitive and tolerant families after *CA2* or *ATP1B* knockdown under nitrite stress. Data were shown as mean ± SD (n = 3). For panels (**A**–**G**), ordinary two-way ANOVA followed by Tukey’s multiple comparisons test was used. For panels (**I**,**J**), Student’s *t*-test was used for the indicated pairwise comparisons. For panels (**H**,**K**,**L**), survival curves were compared using Kaplan–Meier analysis with the log-rank test. Asterisks indicate significant differences between the indicated groups. *p* < 0.05 (*), and *p* < 0.01 (**).

To verify the functional significance of the observed transcriptional differences, RNA interference (RNAi) was performed to silence *CA2* and *ATP1B* genes. Considering that *ATP1B* determines the stability and membrane localization of Na^+^/K^+^-ATPase and *CA2* mediates acid–base buffering to support ion transport, ATP content and Na^+^/K^+^-ATPase (NKA) activity were measured as functional indicators of gill ion-transport capacity. Under nitrite stress, *CA2* exhibited early induction and peaked at 24 h at approximately 12.9-fold that of the dsEGFP control group (*p* < 0.01), whereas *ATP1B* showed delayed induction, peaking at 48 h at approximately 1.9-fold (*p* < 0.01), before declining at 72 h (Figure 6A). Knockdown efficiency was evaluated at 24, 48, and 72 h after nitrite exposure, and both genes remained significantly suppressed at all time points under both control and nitrite-stressed conditions (Figure 6B). Notably, *CA2* knockdown also significantly reduced *ATP1B* expression (*p* < 0.01). Functionally, compared with the nitrite-stressed dsEGFP group, silencing either gene significantly reduced ATP content and NKA activity from 24 to 72 h and further decreased hemolymph pH, particularly at 72 h (*p* < 0.01) (Figure 6C–E). Nitrite accumulation was also aggravated after gene knockdown. Gill nitrite content reached its highest level in the nitrite-stressed dsATP1B group at 72 h (approximately 80.4 mg/mg protein), whereas hemolymph nitrite content reached its highest level in the nitrite-stressed dsCA2 group at 72 h (approximately 546.5 mg/L) and peaked in the nitrite-stressed dsATP1B group at 48 h (approximately 518.8 mg/L) (Figure 6F,G). These changes were accompanied by lower survival during the 96 h nitrite challenge, with final survival rates of 40.0% in *CA2*-silenced shrimp and 36.7% in *ATP1B*-silenced shrimp, compared with 66.7% in the control animals exposed to nitrite (Figure 6H). Family-based validation further confirmed efficient knockdown of *CA2* and *ATP1B* in both the sensitive and tolerant families (Figure 6I,J). Under control conditions, silencing of either gene had negligible effects on survival in both families (Figure 6K). Under nitrite stress, the tolerant family exhibited significantly higher survival than the sensitive family in the dsEGFP control group (70.0% vs. 40.0%). However, knockdown of either *CA2* or *ATP1B* markedly reduced survival in both families, lowering final survival to 20.0% and 23.3% in the sensitive family, and to 30.0% and 36.7% in the tolerant family, respectively (Figure 6L). Although the tolerant family still maintained marginally higher survival after gene knockdown, the survival gap between the two families was substantially narrowed, indicating that disruption of either gene largely abrogated the survival advantage of the tolerant family under nitrite stress. Collectively, these results demonstrate that both *CA2* and *ATP1B* are essential for maintaining ATP-dependent ion transport, acid–base homeostasis, and limiting internal nitrite accumulation, thereby contributing critically to nitrite tolerance in *P. vannamei*.

## 4. Discussion

The present study indicates that variation in nitrite tolerance between families of *P. vannamei* is strongly associated with the capacity of the gill to preserve ion transport, acid–base balance, and energy homeostasis during stress. The tolerant family showed higher survival, lower nitrite accumulation in the gill and hemolymph, milder gill injury, and better maintenance of ATP content, NKA activity, and hemolymph pH under nitrite exposure. These constitutive differences should be regarded as a basal transcriptional context rather than direct evidence for a nitrite-tolerance mechanism. Among the PC2-contributing genes, prohibitin-2 and 39S ribosomal proteins L15 and L9 were associated with mitochondrial maintenance and ribosomal function, respectively [46,47]. Their contribution to PC2 suggests that the two families may differ in basal cellular maintenance and protein-synthesis-related status, which could influence their subsequent response to nitrite stress. However, the functional contribution of these genes to nitrite tolerance remains indirect and requires further validation. Together, these findings indicate that tolerance divergence is more closely associated with the ability to preserve branchial physiological function during exposure, with basal family-specific differences likely acting as a contributory rather than dominant component.

Consistent with this interpretation, the sensitive family exhibited a much broader DEG response, which in this context is more likely to reflect greater physiological disturbance and compensatory demand than a more effective adaptive program. By contrast, oxidative phosphorylation was prominently enriched in the tolerant family, in line with previous omics-based studies linking nitrite tolerance in *P. vannamei* to enhanced energy metabolism [37,38]. The enrichment of oxidative phosphorylation and ribosome-related pathways in the tolerant family was broadly consistent with the PC2-associated genes described above, but this relationship should be regarded as supportive rather than conclusive. Importantly, the tolerant phenotype was not defined by energy metabolism alone, but also by coordinated regulation of membrane transport, proton handling, and acid–base regulatory pathways.

This interpretation is further supported by the family-specific regulation of ion transport- and acid–base-related genes, particularly *CA2*, *ATP1A*, *ATP1B*, and multiple V-type H^+^-ATPase subunits. In crustaceans, the gill is the principal epithelial organ responsible for ion exchange and acid–base regulation and also a major interface for toxicant exchange [30,46]. Carbonic anhydrase, Na^+^/K^+^-ATPase, and V-type H^+^-ATPase are recognized as core components of gill homeostasis [48,49,50,51,52]. In the tolerant family, these transport- and buffering-related genes showed stronger or more sustained induction under nitrite stress and were accompanied by higher NKA activity, higher hemolymph pH, and lower nitrite accumulation in both gill and hemolymph. Collectively, these findings indicate that the tolerant family had a greater capacity to sustain branchial ion transport and acid–base homeostasis during exposure, thereby limiting internal nitrite burden and preserving survival. This interpretation is consistent with previous studies showing that nitrite exposure disrupts hemolymph ion composition, osmolality, and acid–base status while promoting nitrite accumulation in tissues [53,54], and can further impair oxyhemocyanin function and systemic physiology in penaeid shrimp [55].

Classical studies have mainly interpreted nitrite toxicity from the perspective of branchial nitrite entry and toxic load [56,57]. More recent shrimp studies further indicate that transporter-mediated processes contribute to nitrite handling, with *NKCC1* and *AE2* implicated in nitrite influx and hemolymph nitrite regulation under stress [26,32]. Building on this foundation, the present results suggest that the capacity to preserve branchial epithelial homeostasis after exposure may represent an additional determinant of tolerance divergence. In this context, tolerance appears to depend not only on how much nitrite enters the animal but also on how effectively the gill maintains ion transport, acid–base regulation, and physiological stability once exposure has occurred. This view is supported by the present observation that the tolerant family simultaneously showed lower internal nitrite burden, less severe gill injury, and better maintenance of ATP content, NKA activity, and hemolymph pH.

The survival advantage of the tolerant family relies, at least in part, on *CA2*- and *ATP1B*-mediated branchial regulation under nitrite stress. RNAi revealed that silencing either gene decreased survival, ATP content, and NKA activity, exacerbating hemolymph acidification and nitrite accumulation, suggesting that impaired buffering and pump-dependent transport disrupt gill homeostasis and increase internal nitrite burden. These findings align with the known functions of carbonic anhydrase in acid–base balance and Na^+^/K^+^-ATPase in epithelial ion transport [58,59,60]. Despite stronger transcriptional induction of *CA2*, *ATP1B* silencing resulted in a similarly severe physiological phenotype, indicating that transcriptional upregulation does not always reflect functional importance. The reduced *ATP1B* expression following *CA2* silencing further supports coordinated regulation between acid–base buffering and ATP-dependent ion transport. Family-based RNAi validation confirmed that knockdown of either gene significantly diminished the superior survival of the tolerant family under nitrite stress, while exerting minimal effects under control conditions. Collectively, these data demonstrate that *CA2* and *ATP1B* act as functional determinants, rather than passive markers, of family-dependent nitrite tolerance in *P. vannamei*.

Our previous work showed that nitrite-tolerant and nitrite-sensitive families also differ in autophagy- and apoptosis-related responses under nitrite exposure [23,38]. The present study adds an upstream physiological dimension to that framework, suggesting that gill homeostatic regulation is an upstream determinant of family-dependent tolerance, whereas downstream cellular stress responses more likely reflect the consequences of internal nitrite burden. This interpretation also helps explain why the sensitive family exhibited more extensive transcriptomic changes despite lower nitrite tolerance. A larger transcriptional response in this context is more indicative of greater homeostatic disruption and a higher demand for compensatory mechanisms, rather than reflecting superior adaptive capacity. Previous studies likewise showed that nitrite stress can impair hemolymph physiology, respiratory status, and energy metabolism in shrimp [53,54,55,61].

Several limitations should nevertheless be acknowledged. Although oxidative phosphorylation-related pathways were enriched in the tolerant family and ATP content was higher, mitochondrial function and ATP production efficiency were not directly assessed. Moreover, RNAi efficiency for CA2 was confirmed only at the transcript level, without direct validation at the protein or carbonic anhydrase activity level, which weakens the CA2-specific functional interpretation. In addition, a PBS-injected control was not included in the present RNAi design, and thus the potential contribution of injection-related physical stress could not be fully excluded. Specific nitrite entry routes also remain unresolved, and broader downstream endpoints, such as immune status or microbiota stability, were not examined. Future work should therefore integrate direct measurements of mitochondrial performance, protein- or enzyme-level validation of key transport-related regulators, transporter-specific nitrite flux, and systemic downstream responses to clarify how energetic support, branchial transport, and whole-animal stress outcomes are mechanistically coupled during nitrite exposure.

## 5. Conclusions

**Figure 7:**
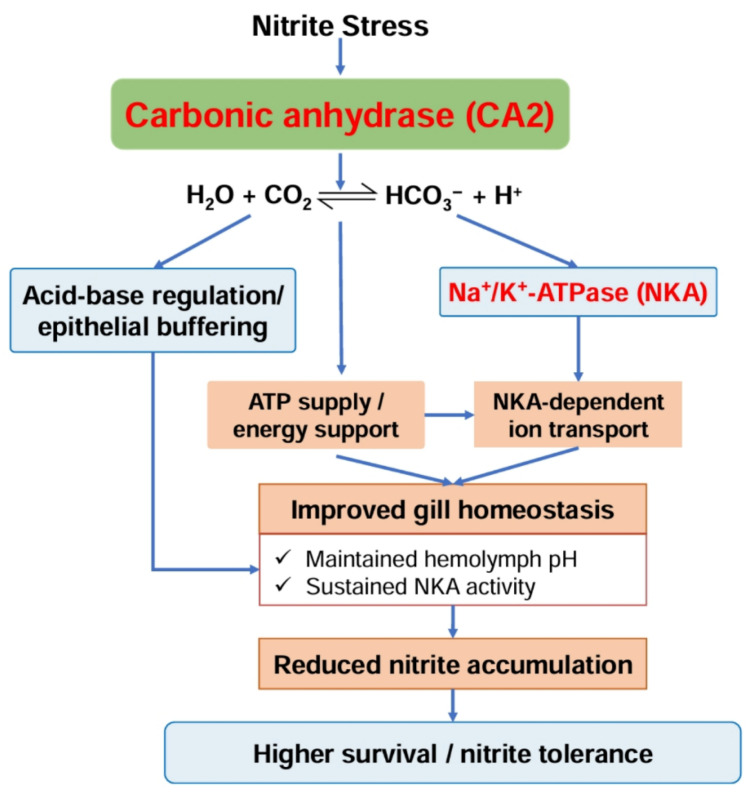
Proposed mechanism of CA2-mediated branchial regulation underlying nitrite tolerance in *P. vannamei*. Under nitrite stress, enhanced CA2-associated generation of HCO_3_^−^ and H^+^ promotes branchial acid–base regulation and epithelial buffering, while supporting Na^+^/K^+^-ATPase (NKA)-dependent ion transport and ATP/energy supply. Together, these responses improve gill homeostasis, as reflected by maintained hemolymph pH and sustained NKA activity, thereby reducing nitrite accumulation and ultimately enhancing survival under nitrite stress. Red text indicates the key enzymes involved in CA2-associated acid–base regulation and NKA-dependent ion transport.

In conclusion, the present study demonstrates that family-dependent nitrite tolerance in *P. vannamei* is primarily determined by the capacity of the gill epithelium to sustain ion transport under nitrite stress, thereby stabilizing acid–base homeostasis and reducing internal nitrite accumulation (Figure 7). Thus, enhanced branchial ion transport, rather than a generalized stress response, represents a central mechanism of nitrite tolerance in shrimp. These findings provide a mechanistic basis for understanding tolerance divergence and for the selective breeding of nitrite-tolerant lines.

## Data Availability

The RNA-seq data generated in this study are available in the Genome Sequence Archive (GSA) under accession number PRJNA1427949.

## Supplementary Materials

The following supporting information can be downloaded at: https://www.mdpi.com/article/10.3390/ani16111638/s1. Figure S1: Time-dependent comparison of median lethal concentration (LC50) between nitrite–sensitive and nitrite–tolerant *P. vannamei* families under nitrite stress; Figure S2: KEGG enrichment analysis of DEGs in nitrite–sensitive and nitrite–tolerant *P. vannamei* families; Table S1: DEGs in gills responded to nitrite stress in sensitive family; Table S2: DEGs in gills responded to nitrite stress in tolerant family; Table S3: DEGs in gills between tolerant and sensitive families under healthy conditions; Table S4: DEGs in gills between tolerant and sensitive families under nitrite stress.

## Abbreviations

The following abbreviations are used in this manuscript:

RNA	Ribonucleic Acid
dsRNA	Double-Stranded RNA
DNA	Deoxyribonucleic Acid
cDNA	Complementary Deoxyribonucleic Acid
PCR	Polymerase Chain Reaction
qPCR	Real-Time Quantitative PCR
DEGs	Differentially Expressed Genes
GO	Gene Ontology
KEGG	Kyoto Encyclopedia of Genes and Genomes
PCA	Principal Component Analysis
mg	Milligram
h	Hour
min	Minute
μL	Microliter
L	Liter
s	Second
PBS	Phosphate Buffered Saline
mmol	Millimole
mL	Milliliter
RNAi	RNA Interference
TPM	Transcripts per million
FDR	False discovery rate
CA2	Carbonic anhydrase 2
ATP1A	Na^+^/K^+^-ATPase α subunits
ATP1B	Na^+^/K^+^-ATPase β subunits
NKA	Na^+^/K^+^-ATPase
VATP-A	V-type proton ATPase subunit A
VATP-C	V-type proton ATPase subunit C
VATP-D	V-type proton ATPase subunit D
VATP-E	V-type proton ATPase subunit E
VATP-G	V-type proton ATPase subunit G
VATP-H	V-type proton ATPase subunit H
VATP-S1	V-type proton ATPase subunit S1
βCA1	Beta-type Carbonic Anhydrase 1
NBC3	Na^+^-HCO_3_^−^ Cotransporter 3
NHE9B2	Na^+^/H^+^ Exchanger 9B2
EGFP	Enhanced green fluorescent protein
LC_50_	The median lethal concentration

## Appendix A

**Table A1:** Primer sequences used in this paper.

Primers	Sequence (5′ → 3′)
For qRT-PCR	
qActin-R	TGAAGATCCTGACGGAGCGT
qActin-F	GAACCTCTCGTTGCCGATG
qCA2-F	AATCAGCGGCGGAGAGTTGG
qCA2-R	AATGCCCAACACAGCAAGGC
qATP1B-F	CATCCAGCATTGGGTCGAGT
qATP1B-R	AGGAGATTCGCGGTTGTAGC
qATP1A-F	GCCTTCCTTTCCTACACCCC
qATP1A-R	CCAACCACCAGGGTTCCTAC
qVATP-C-F	CAAACTCCATGCCCGTGAGA
qVATP-C-R	TCAACAAACACACGGATGGC
qVATP-H-F	GACGAGCCACAGGTATCAGG
qVATP-H-R	TGAGGAAGGGGCTCCACATA
qVATP-E-F	AGGCCCAGGAGTCCCATATT
qVATP-E-R	GGCCATAGAATGCTCCACCA
qCLCA-R	TGTACGGCCTAGACGAAGGA
qCLCA-F	TAGGTGACGTTGAAGGCGAC
For dsRNA	
EGFP-F	CCCGACCACATGAAGCAGCA
EGFP-R	GTAGTGGTTGTCGGGCAGCA
EGFP-T7F	TAATACGACTCACTATAGGGCCCGACCACATGAAGCAGCA
EGFP-T7R	TAATACGACTCACTATAGGGGTAGTGGTTGTCGGGCAGCA
dsCA2-F	CTCCTGGGCGGTCTGGTC
dsCA2-R	TGGACAGGGCGGTAGTTGTCT
dsCA2-T7F	TAATACGACTCACTATAGGGCTCCTGGGCGGTCTGGTC
dsCA2-T7R	TAATACGACTCACTATAGGG TGGACAGGGCGGTAGTTGTCT
dsATP1B-F	ATCGACTGGGTTCCTGATGTG
dsATP1B-R	AGCAACAATAGGTGGCAGGT
dsATP1B-T7F	TAATACGACTCACTATAGGGATCGACTGGGTTCCTGATGTG
dsATP1B-T7R	TAATACGACTCACTATAGGGAGCAACAATAGGTGGCAGGT
