# Supplementary material for: Carbonic Anhydrase 2 and Na+/K+-ATPase Mediate Family-Dependent Nitrite Tolerance via Modulating Branchial Ion Transport and Acid–Base Balance in Penaeus vannamei
Source: Animals (Basel). 2026 May 27;16(11):1638. doi: 10.3390/ani16111638 (PMC13255585; doi:10.3390/ani16111638)
Supplement: Supplementary file 1 [file animals-16-01638-s001.zip › Supplementary Figures.pdf]

Supporting Information

Supplementary Figure

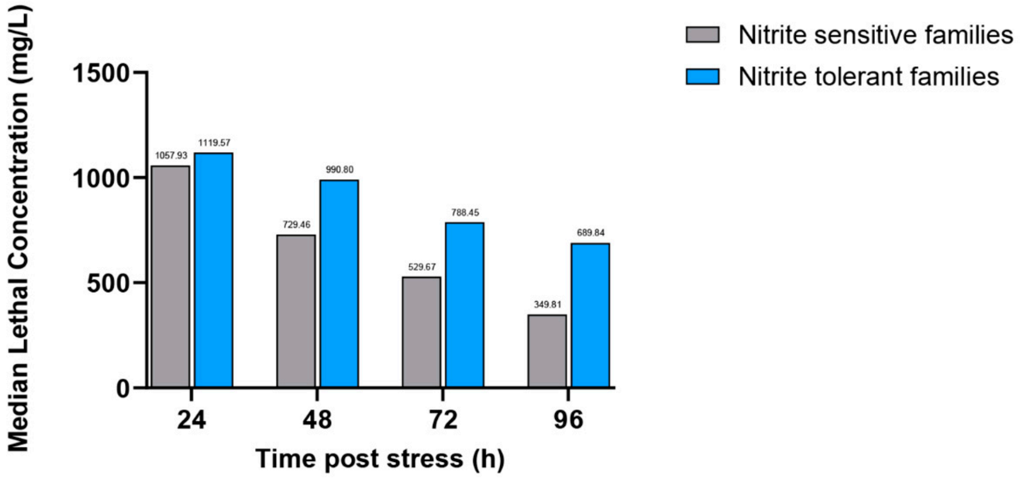

Figure S1 Time-dependent comparison of median lethal concentration (LC_{50}) between nitrite-sensitive and nitrite-tolerant *P. vannamei* families under nitrite stress. Estimated LC_{50} values of nitrite in nitrite-sensitive and nitrite-tolerant *P. vannamei* families at 24, 48, 72, and 96 h after nitrite exposure. Values labeled above the bars indicate the estimated LC_{50} at each time point.

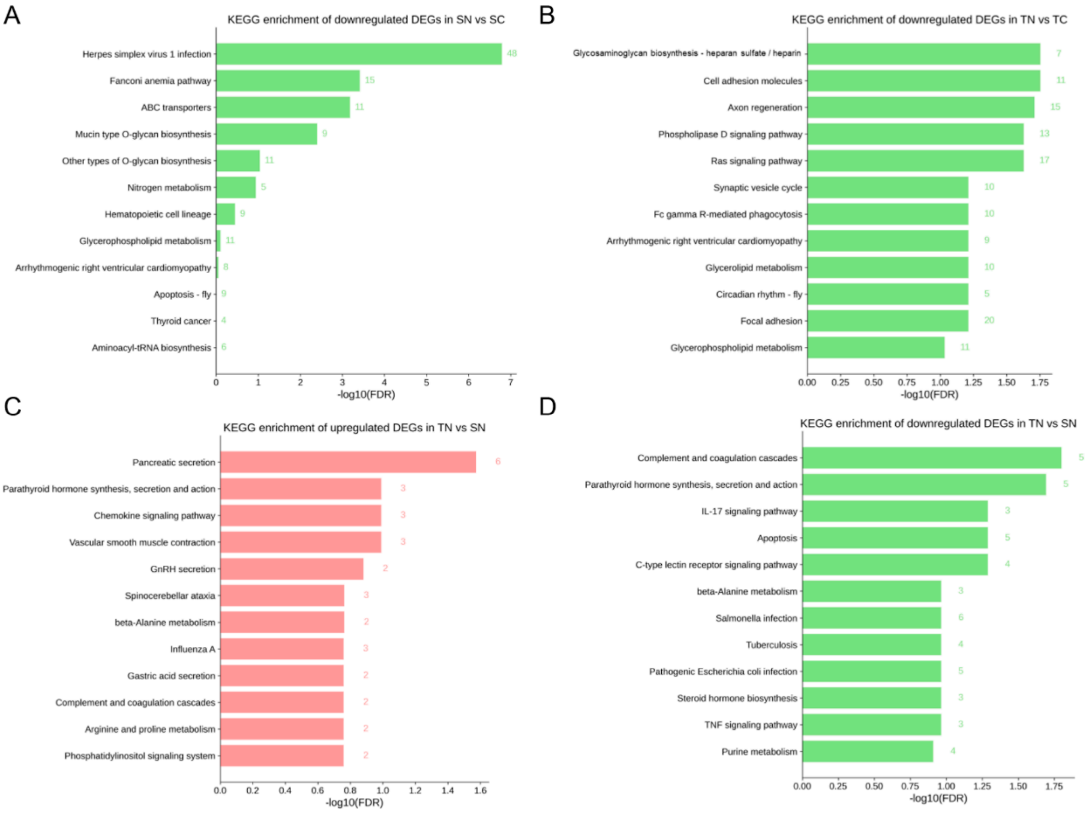

Figure S2 KEGG enrichment analysis of DEGs in nitrite-sensitive and nitrite-tolerant *P. vannamei* families. (A) Downregulated DEGs in SN vs SC. (B) Downregulated DEGs in TN vs TC. (C) Upregulated DEGs in TN vs SN. (D) Downregulated DEGs in TN vs SN. The x-axis shows −log10(FDR), and the numbers beside the bars indicate the number of enriched DEGs in each pathway.
